# Cholangioscopy-assisted endoscopic ultrasound-guided gastroenterostomy

**DOI:** 10.1055/a-2808-6791

**Published:** 2026-03-11

**Authors:** Shan-Shan Hu, Wei-Hui Liu

**Affiliations:** 1 89669 Department of Gastroenterology and Hepatology, Sichuan Provincial Peopleʼs Hospital, School of Medicine, University of Electronic Science and Technology of China, Chengdu, China

Endoscopic ultrasound-guided gastroenterostomy (EUS-GE) is an advanced technique primarily used to treat both benign and malignant gastric outlet obstruction 1 2 . This approach overcomes the limitations of traditional duodenal stent placement and surgical procedures 3 , providing an effective alternative therapy. A critical step in the procedure is passing the guidewire through the stricture into the distal bowel segment. Failure at this stage prevents the successful completion of EUS-GE. Recently, we developed a new technical approach and stricture-crossing strategy.

**Fig. 1:**
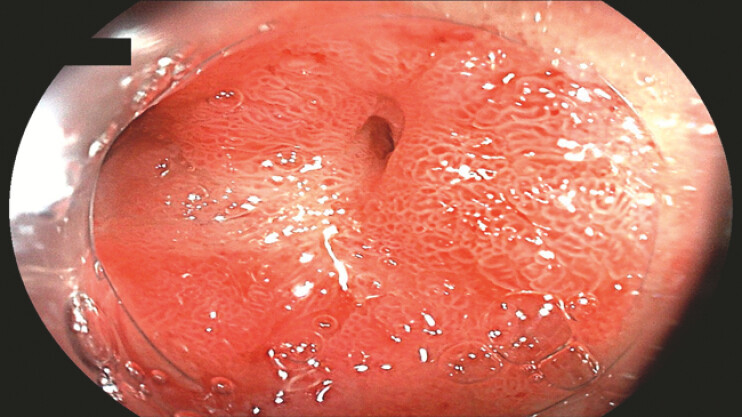
Malignant duodenal obstruction.

**Fig. 2:**
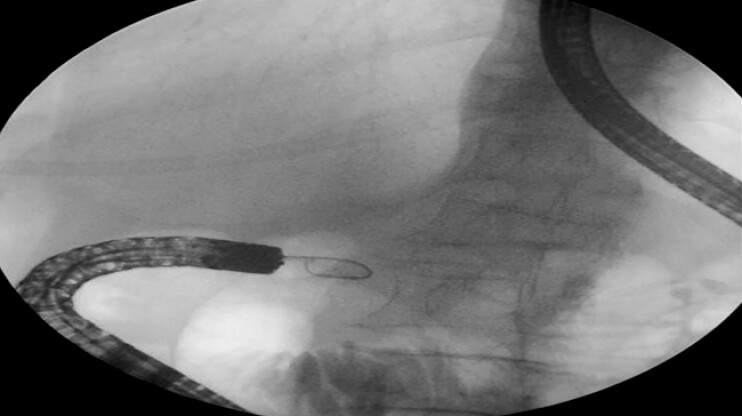
The guidewire failed to pass through the narrowed segment.

**Fig. 3:**
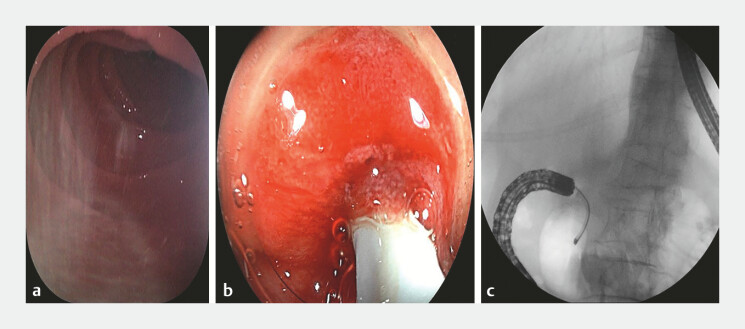
A cholangioscope successfully traversed the stricture. **a** The cholangioscope successfully traversed the stricture and entered the distal intestine. **b** Endoscopic imaging. **c** X-ray confirmed that the cholangioscope had successfully passed through the stricture.

**Fig. 4:**
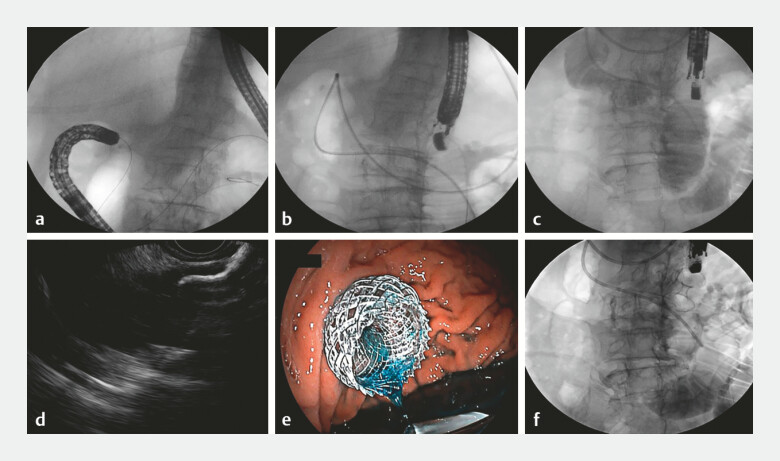
EUS-GE procedural steps. **a** The guidewire was successfully advanced into the jejunum. **b** The nasobiliary tube was accurately inserted into the jejunum over the guidewire. **c** X-ray imaging localized the target intestinal segment. **d, e** Completion of gastro-jejunal anastomosis. **f** The stent was confirmed to be in proper position under X-ray. EUS-GE, Endoscopic ultrasound-guided gastroenterostomy.

**Fig. 5:**
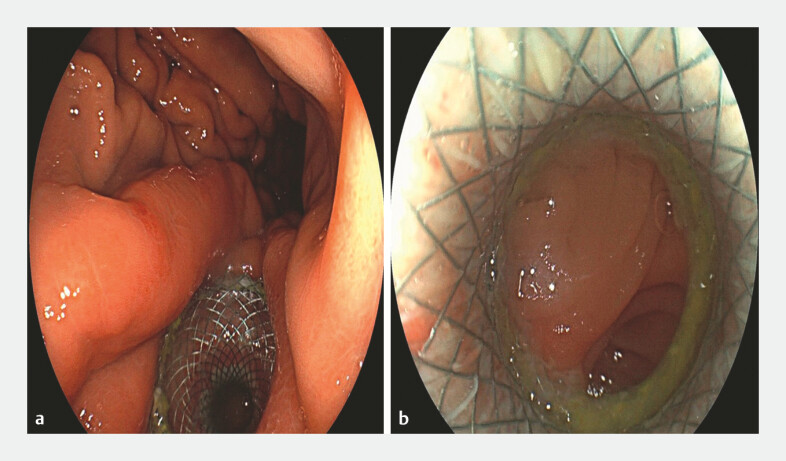
Postoperative gastroscopy. **a** The stent remained in a proper position. **b** Stent patency was maintained.

We present the case of a 65-year-old male patient with gastric outlet obstruction caused by duodenal cancer with systemic metastasis, who was deemed unsuitable for surgical resection ( Fig. 1 ). EUS-GE was performed to relieve the obstruction. Despite repeated attempts using a disposable sphincterotome and a guidewire to traverse the duodenal stricture ( Fig. 2 ), the narrowed segment could not be crossed. We then employed a novel combination of colonoscopy and cholangioscopy. The colonoscope was advanced to the site of the duodenal obstruction, and a cholangioscope was inserted through the colonoscope’s working channel into the stricture. Under direct visualization, a pathway was identified and quickly navigated across the stricture into the distal intestine ( Fig. 3 ). The nasobiliary tube was inserted along the guidewire and pushed into the jejunum. Methylene blue mixed with contrast saline solution was injected, and the target bowel location was confirmed by both endoscopic ultrasound and fluoroscopy. A 15-mm cautery-enhanced lumen-apposing metal stent was then punctured directly through the gastric wall into the jejunum, completing the gastro-jejunal anastomosis ( Fig. 4 ). Follow-up gastroscopy on postoperative day 3 demonstrated good stent patency ( Fig. 5 ). Follow-up was conducted at 1, 3, and 6 months postoperatively. The patient maintained a liquid diet, with GOOSS scores stabilized at 2–3 points, requiring no further intervention and without complications ( Video 1 ).

We developed a new technical approach and stricture-crossing strategy, cholangioscopy-assisted endoscopic ultrasound-guided gastroenterostomy.Video 1

Conventional methods often encounter significant difficulties. Guidewires are prone to require repeated trial-and-error maneuvers, which are frequently unsuccessful in traversing strictures. Moreover, ultra-fine endoscopes, while highly flexible, lack the necessary support, making manipulation cumbersome. In contrast, the colonoscope’s working channel provides stable support for the cholangioscope. With direct visualization, the cholangioscope can accurately identify and traverse the stricture. This innovative technique has considerable clinical value and holds strong potential for broader adoption in medical practice.

Endoscopy_UCTN_Code_TTT_1AS_2AK
